# An evidence-informed, community-engaged approach to designing a large-scale, impact-oriented research funding initiative to foster the implementation of transformative integrated care: a multi-methods qualitative study

**DOI:** 10.1186/s43058-025-00760-7

**Published:** 2025-08-15

**Authors:** Nida Shahid, Jessica Nadigel, Rhonda Boateng, Richard H. Glazier, Meghan McMahon

**Affiliations:** 1 CIHR Institute of Health Services and Policy Research, 2075 Bayview Avenue, Toronto, Ontario, M4N 3M5 Canada; 2 Institute of Health Policy, Management and Evaluation, , 155 College Street, Suite 425, Toronto, Ontario M5T 3M6, University of Toronto, 155 College Street, Suite 425, Toronto, Ontario, M5T 3M6 Canada; 3 Institute for Clinical Evaluative Sciences, V Wing, V1-06, 2075 Bayview Avenue, Toronto, Ontario, M4N 3M5 Canada; 4 MAP Centre for Urban Health Solutions, St. Michael’s Hospital, 30 Bond Street, Toronto, Ontario, M5B 1W8 Canada; 5 University of Toronto Department of Family and Community Medicine, 500 University Avenue, 5th Floor, Toronto, Ontario, M5G 1V7 Canada; 6 Dalla Lana School of Public Health, University of Toronto, 155 College Street, 6th Floor, Toronto, Ontario, M5T 3M7 Canada

**Keywords:** Health services and policy research, Community-engaged, Implementation science, Integrated care

## Abstract

**Background:**

Integrated care is a promising strategy to advance system transformation, care coordination, equity, and better health outcomes. Health services and policy research can drive evidence-informed health system improvements but is often underutilized. To optimize the relevance and impact of integrated care research as a transformative lever for better health and system outcomes, the Canadian Institutes of Health Research’s Institute of Health Services and Policy Research (CIHR-IHSPR) designed a large-scale, evidence-informed, community-engaged research funding initiative. This paper outlines the approach and methods used by CIHR-IHSPR and describes how they informed the design and development of Transforming Health with Integrated Care (THINC), a large-scale, impact-oriented research funding initiative that promotes the adoption and proliferation of integrated care in Canada.

**Methods:**

A multi-method qualitative, community-engaged approach was used to inform the design of a research funding strategy. Key features of the approach included multiple evidence inputs (retrospective and prospective information from primary [key informant interviews, focus groups, and a workshop] and secondary [CIHR funding data and literature review] sources), pan-Canadian reach of community engagement, involvement of diverse interest-holders, iterative data collection and analysis, and a commitment to identifying shared priorities through a community-engaged process.

**Findings:**

There was consensus across the evidence inputs that implementing, adapting, and scaling evidence-informed integrated care interventions is crucial for real-world impact. Strategies found important for improved research relevance and impact include implementation science, rapid response, embedded research, and knowledge mobilization, along with key initiative design elements such as co-leadership, cross-jurisdictional and interdisciplinary teams, and a focus on the Quintuple Aim. Priority populations were also identified for maximizing the potential benefit and impact of the research. These findings informed the design of THINC, resulting in a multi-program initiative aligned to a shared goal of evidence-informed integrated care transformation. A collaborative design approach fostered shared objectives, commitment from multiple partner organizations, and resources to increase the initiative’s size and scope.

**Conclusions:**

The study demonstrates the feasibility of using an evidence-informed, community-engaged approach and the influence and benefits of the approach in designing a large-scale research funding initiative that aims to be transformational and impactful.

**Supplementary Information:**

The online version contains supplementary material available at 10.1186/s43058-025-00760-7.

Contribution to the literature

- This paper demonstrates a health research funder’s evidence-informed and community-engaged approach to designing a large-scale research funding initiative grounded in shared objectives and a commitment to research strategies that advance equity and real-world impact, contributing to the emerging field of the science of funding for research impact.
- The study provides evidence-informed strategies grounded in collaboration, engagement of diverse perspectives, and a community-engaged approach to designing a transformative research funding initiative, and it illustrates the impact of these strategies on the resulting initiative’s relevance, scope, and potential impact.

## Introduction

Health systems worldwide strive to achieve the Quintuple Aim for healthcare improvement (improved cost, efficiency, equity, patient and provider experiences) [1–4]. Integrated care offers a promising strategy to advance system transformation and better health outcomes [5–8]. Health systems and policy research (HSPR) funders are uniquely positioned to stimulate evidence-informed health system improvement, contribute by setting research priorities, strengthening capacity, and advancing knowledge mobilization (KM) [9–11].

Research funding strategies are crucial for improving system performance and enhancing research impact [12–14]. Growing interest in research impact stems from a variety of factors: budget constraints requiring demonstrated returns on investments [15], persistent suboptimal system performance and worsening inequities [16, 17]. To improve the potential impact of the HSPR funding towards achieving the Quintuple aim, the *Canadian Institutes of Health Research – Institute of Health Services and Policy Research* (CIHR-IHSPR) designed a large-scale research funding initiative using an evidence-informed, community-engaged approach. This paper describes the methods used to design the priority-focused, impact-oriented research funding initiative and demonstrates the results of those methods in the creation of the resulting initiative.

### Canada’s need for transformative integrated care

Canada’s health care systems underperform compared to peer countries, especially in integration, care coordination, and digital health [18]. With 13 provincial/territorial systems and federal programs, services remain hospital-centric and siloed across sectors [19], struggling to serve a growing population with complex care needs [20], COVID-19 intensified existing system deficiencies and inequities [21, 22], while the health workforce crisis and inadequate primary care access necessitate transformational change [23–27].

Integrated care is critical to delivering higher quality of care, better outcomes and experiences, and at a reduced cost [37] and has been identified as a core priority for many provinces and territories in Canada and other countries [38]. Integrated care, also known as ‘*continuity of care’*, ‘*transitions of care’*, or ‘*coordinated care’,* bridges acute, primary, and community and social care services for better patient experience and outcomes [23, 28–32] improving coordination, efficiency, and, equity. Canadian models include Ontario Health Teams (Ontario) [33], Quebec’s Family Medicine Groups [34], and formerly Alberta’s Strategic Clinical Networks, representing Canada’s first province-wide fully integrated system bringing together 12 formerly separate health entities [35]. However, limited implementation and evaluation contributes to suboptimal system performance [36–38]. The transformation of Canada’s health care systems towards integrated care can facilitate its goal to achieve the Quintuple Aim [39].

### The role of research funders and opportunity within health services and policy research

Research funding organizations connect knowledge to practice, aligning programs with system priorities [40–42]. Globally, funders seek to invest in research generating real-world impact [43–45], advancing the science of research funding (i.e., how to fund for impact), and impact assessment [43, 46]. Organizations like the *Research-on-Research Institute,* the *Transforming Evidence Funders Network,* and the *Canadian Health Services and Policy Research Alliance* (CHSPRA) work to improve how research is funded and evaluated for better impact [47–49].

Since 2000, the CIHR-IHSPR has funded solution-oriented research, supporting initiatives to improve the organization, regulation, management, financing, and delivery of healthcare services for better health and quality of life (Canadian Institute of Health Services and Policy Research, 2021). A 2019–2020 pan-Canadian strategic planning exercise by CIHR-IHSPR engaged diverse interest-holders^1^ who consistently identified integrated care as a top priority for research investment and action (Canadian Institutes of Health Research—Institute of Health Services and Policy Research, 2021). This paper outlines CIHR-IHSPR’s evidence-informed, community-engaged approach to designing the Transforming Health with Integrated Care (THINC), a large-scale research funding initiative to advance transformative integrated care, aiming to understand the essential elements of integrated care, priority areas for investment, effective funding mechanisms and opportunities for ongoing engagement. That is, THINC was not developed a priori of the engagement.

## Methods

**Fig. 1:**
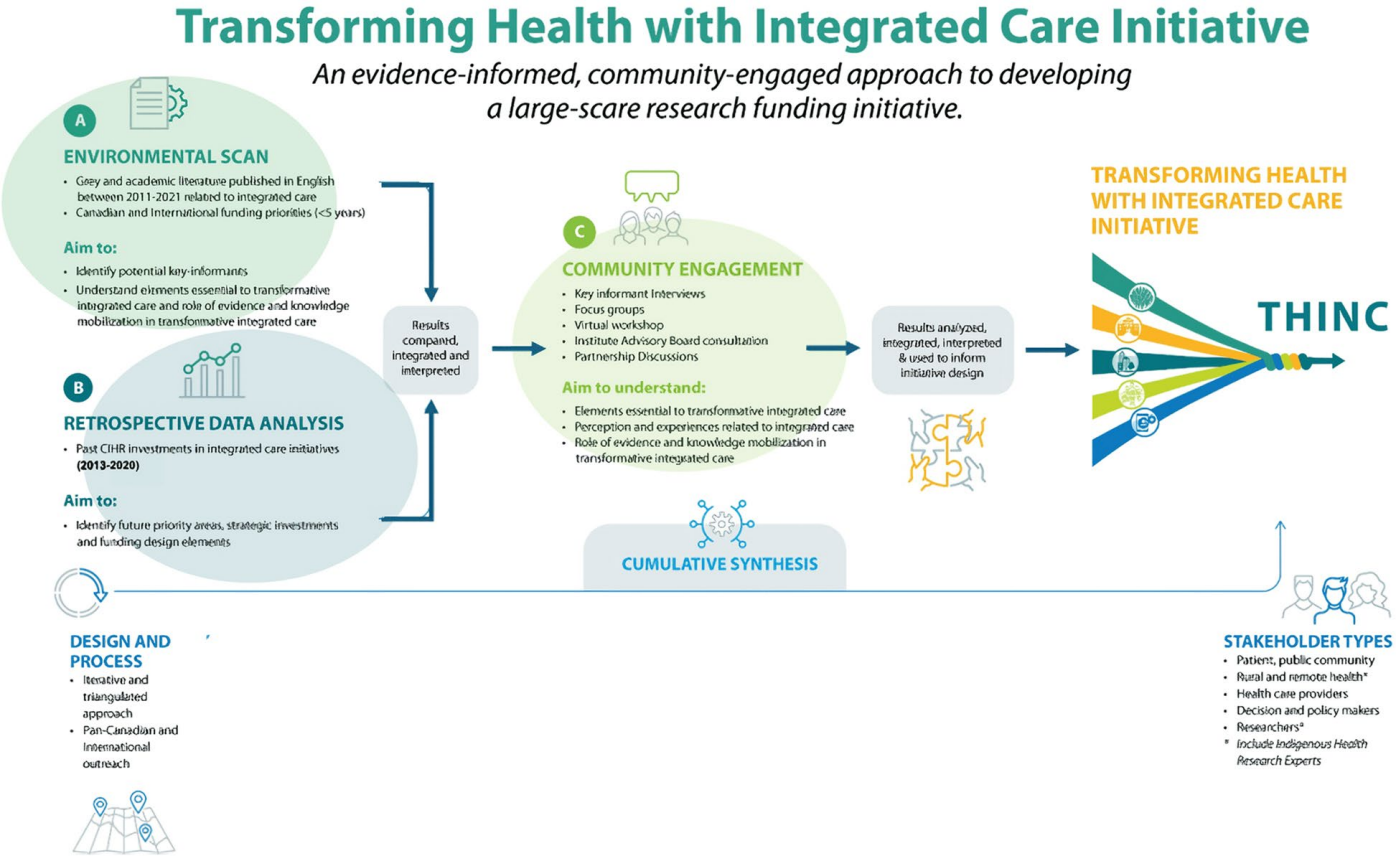
THINC: overview of an evidence-informed, community-engaged approach to developing a large-scale research initiative

CIHR-IHSPR adopted a qualitative, iterative, multi-method approach to design the research funding initiative: (i) environmental scan of peer-review and grey literature, (ii) a retrospective analysis of historical research funding investments, and (iii) community engagement (see Fig. 1). These phases were iterative rather than sequential, continuously informing one another throughout the process.

### Data collection

#### Environmental scan

An environmental scan of the peer-reviewed and grey literature was conducted between May and June 2021 to understand definitions and elements of transformative integrated care, the role of research and KM, and how funding design can support integrated care. The scan provided foundational insights for designing the community engagement activities.

Peer-reviewed and grey literature published in English between 2011–2021 were searched using Medline/PubMed, Google Scholar, coupled with hand searches of relevant organizational websites. The time frame was selected to capture any long-term research funding cycles (e.g., Horizon 2020 has a long-term budget from 2014–2020 [50].

Concepts related to integrated care were explored using keywords: ‘integrated care’, ‘coordinated care’, ‘transitions in care’ and combined with terms related to transformative approaches (e.g., health system transformation). Boolean operators (AND, OR) were used to refine searches (e.g., “integrated care” OR “coordinated care” AND “knowledge mobilization” to explore the subthemes including definitions, essential elements, the role of research, funding design.

Websites of key organizations including international and Canadian provincial and federal funding agencies were also searched to understand the extent to which integrated care was prioritized for research investment and, if so, the funding design elements used. Sources were selected for review based on relevance to study topic.

#### Retrospective analysis

The retrospective analysis aimed to assess CIHR’s historical research investments related to integrated care (2013–2019). This helped identify previous funding patterns, priority areas for investments, and the alignment of past programs with the emerging goals of the new initiative.

The analysis focused on key programs known to encompass integrated care and related research, including the *Strategy for Patient-Oriented Research* (SPOR) [51] and its Primary and Integrated Health Care Innovations Network [51], Community-based Primary Health Care (CBPHC) initiative (CIHR Community-Based Primary Health Care, 2017), and Transitions in Care (TiC) initiative [52]. Additionally, a keyword search was conducted within CIHR’s funded research database, specifically using keywords ‘continuity’, ‘transitions’ and/or ‘integration of care’. This search aimed at identifying potentially relevant research projects supported through other strategic and open investigator-driven programs. Relevance was independently assessed by two CIHR-IHSPR team members, with discrepancies discussed and resolved with the input of a third team member. Descriptive statistics were used to quantify the investment levels across different areas, while thematic analysis was used to categorize the different research areas (e.g., patient-centered care, implementation) and design elements (e.g., embedded research, rapid response).

Data was extracted using Excel and organized into broad categories including project topics, funding amounts, and funding design elements (e.g., embedded research, leadership model).

#### Community engagement

Community engagement was conducted to collect insights from interest-holders with expertise and/or lived/living experiences with integrated care. This phase was crucial for shaping the initiative’s scope, funding objectives and design elements. Engagement activities were conducted between June-October 2021 and included:

- Key-informant interviews (KII)
- Focus groups
- Virtual workshop
- Partner engagements

Participants included researchers, healthcare providers, decision and policy makers, people with lived and living experience^2^ (PWLLE), and potential partner organizations (i.e., funding partners and action partners [service delivery, policy organizations] from Canada and internationally. Participants provided informed consent and were given the option to opt out of the study at any time.

#### Recruitment

Participants were identified using purposive sampling through referrals by CIHR-IHSPR’s network (e.g., advisory board, funded researchers) and online searches for individuals with relevant expertise. Snowballing sampling was also used to broaden reach and ensure diversity in interest-holder type, professional expertise (e.g., researcher, providers, patient/caregivers), geographic location (e.g., representation from all provinces, territories, including rural and remote areas), and other demographic characteristics (e.g., gender, career stage) [53]. Informants were recruited until data saturation was reached [54]. Verbatim transcriptions were made of all interviews, focus groups, workshop discussions, and partner meetings. At the start of each interview and focus group, participants were reminded of the study aims and that their participation was voluntary, they could opt-out at any time, and all data would be anonymized. At least two CIHR-IHSPR team members joined each interview and focus group discussion: one as the interview, the other as note-taker.

#### Interview and focus group guide development

KII and focus group guides were developed based on insights from the environmental scan and goals of the funding initiative. The guides were semi-structured and tailored to the different participants groups (e.g., providers, researchers, PWLLE) to ensure the questions were contextually relevant and designed to encourage open-ended discussion.

#### Key informant interviews

Participants were invited by email for a 45–60 min semi-structured interview conducted on Zoom. Interview guides were shared in advance (Appendix 1). Interviews were recorded with permission for transcription of data.

#### Focus groups

Focus groups were held with three priority communities: PWLLE and members of the public, healthcare providers, and the rural health research and practice community, including Indigenous health experts. Each session lasted 60–90-min, were held on Zoom and recorded with permission for transcription and data analysis. A discussion guide (Appendix 2) was shared in advance.

#### Virtual workshop

A virtual workshop at the 1 st North American Conference on Integrated Care (October 2021) was hosted by CIHR-IHSPR. Panelists were invited based on their expertise and role within the health care system (i.e., patient partner, healthcare provider, health services and policy researcher, health system decision-maker). A discussion guide (Appendix 3) was co-developed with the panelists to facilitate discussion related to integrated care and the role of research in advancing transformative integrated care. The workshop provided an important opportunity to engage with and learn from international perspectives.

#### Partner engagements

Partner engagement refers to our process of identifying and consulting with two types of organizations: (i) research funding organizations as potential partners interested in informing the design and/or support the initiative and (ii) action organizations, such as government and service delivery organizations involved in integrated care service delivery and/or policy, to inform our understanding of system priorities and evidence needs related to integrated care.

Potential partners were identified through CIHR-IHSPR’s network and included CIHR institutes and initiatives (CIHR comprises 13 institutes and several initiatives like SPOR), pan-Canadian health organizations, provincial and territorial health departments and authorities, and federal, provincial, and international health research funding organizations with interests related to integrated care. Between August 2021 and February 2022, partner engagements were conducted virtual (30–60 min) using Zoom with potential partners to discuss the proposed funding initiative, garner feedback on research priorities, funding design elements, and explore potential financial and/or in-kind partnership opportunities. Meetings were recorded with permission for transcription and analysis.

#### Institute advisory board

CIHR-IHSPR’s Institute Advisory Board (IAB) provided strategic guidance throughout the development of the initiative. The IAB includes 14 national and international representatives of public, private, and non-profit sectors including the research community, health practitioners, decision-makers and PWLLE. Bi-annual meetings between CIHR-IHSPR and IAB facilitated input and feedback and ensured that the initiative aligned with CIHR-IHSPR’s strategic priorities.

### Data analysis

Excel was used to organize the data and report on emerging patterns [55]. Data from all sources (environmental scan, retrospective analysis and community engagement) were analyzed to identify emerging themes, patterns and insights relevant to study needs.

Transcripts from interviews, focus groups, workshop discussions, and partner meetings were analyzed using a thematic analysis approach. This process involved familiarization with the data, generating initial codes and refining the codes into broader themes based on recurring concepts. For example, data describing need for continuously monitoring and improving (system) performance or having effective feedback (mechanisms) for providers was thematically coded as ‘monitoring and evaluation’. The resulting themes were reviewed and discussed with the CIHR-IHSPR team to ensure consensus prior to continuing data analysis. To prevent any conflicts of interest and unfair advantage among participants as potential applicants, only the confirmed funding partners were engaged for input in the final version design of the initiative.

The partnership discussions were held concurrently with parts of the community engagement, ensuring all insights were gathered and synthesized in parallel.

### Triangulation and bias assessment

Insights from the community engagement were integrated with findings from the environmental scan and retrospective analysis to refine funding objectives, priorities and key design elements.

The iterative approach allowed for ongoing feedback loops and helped ensure the funding strategy was not only evidence-informed but also reflective of the community perspectives that emerged throughout the engagement process.

The authenticity of qualitative information was ensured by the CIHR-IHSPR team conducting in-depth discussions to select the sampling approach and participant pool, aiming for a diverse representation (e.g., geography, demographic variables, interest-holder type). To further enhance the validity of the findings, source and method triangulation were used. This approach involved comparing information from different sources and methods (e.g., interviews, focus groups, and environmental scan) to assess whether the emerging themes were consistent. The use of multiple methods helped inform a more comprehensive understanding of key enablers to achieve transformative integrated care and the strategies research funders can use to facilitate and enable transformative research [56]. Methods were intentionally tailored (e.g., individual interview vs. focus group discussions) in consideration of time, participant availability, and nature of discussion (e.g., integrated care in the rural health context vs. engagement with patient and public communities). These adjustments ensured that each type of engagement was well-suited to the context in which it took place or adapted based on the participant type (e.g., rural health challenges with integrating care vs. provider perspectives on integrating care). The guiding questions were also tailored based on the type of participant and engagement.

Strategies to minimize potential social desirability bias included engagement activities facilitated or led by a research team member without a senior institutional position, allowing for a more balanced power dynamic in certain discussions and inviting candid insights.

## Findings

CIHR-IHSPR used an evidence-informed, community-engaged approach to inform the development of a large-scale strategic research funding initiative for transformative integrated care. This study identified three main findings. First, evidence suggests the need to shift the focus from developing new models of integrated care to implementation of existing, proven models. Second, funded research needs to align with achieving the Quintuple Aim and priority populations that have the greatest potential benefit from transformative integrated care. Third, transformative integrated care requires diverse and shared leadership in addition to cross-sectoral collaboration. A brief overview of the findings from each study approach is described next, followed by a description of the main study findings.

### Community engagements

The community engagements helped corroborate the essential elements identified in the literature and shed light on priorities within integrated care, the potential value-add of research investment, the research strategies and design elements perceived as key to maximizing the value and impact of research investment. More than 100 individuals across different interest-holder groups and regions were involved in community engagement (see Table 1). The initiative scope, aim and design elements were shaped by the findings of the community engagements, as illustrated below.

**Table 1:** Overview of community engagements

Community Engagement Strategies*	*Sample Size (n)*	*Stakeholder Types*	*Geography*	*Sex*
Key informant interviews	14	• Researcher (*n* = 6) • Decision maker (*n* = 3) • Healthcare provider (*n* = 2) • Researcher/Healthcare provider (*n* = 1) • Decision maker/Healthcare provider (*n* = 1) • PWLLE (*n* = 1)	• Ontario (*n* = 6) • International (*n* = 3) • British Columbia (*n* = 2) • Alberta (*n* = 1) • Nova Scotia (*n* = 1) • Northwest Territories (*n* = 1)	• Female (*n* = 7) • Male (*n* = 7)
Focus Groups	3 (8–10 per group)	• PWLLE and Public Communities • Healthcare providers • Rural health community	• Not available†	• Female (*n* = 14) • Male (*n* = 11)
Workshop	77	• Researchers (*n* = 22) • Policy (*n* = 7) and decision makers (*n* = 15) • Patients (*n* = 6) and caregivers (*n* = 11) • Healthcare providers (*n* = 16)	• Ontario (*n* = 67) • Alberta (*n* = 2) • International (*n* = 4) • British Columbia (*n* = 1) • Nova Scotia (*n* = 1) • Northwest Territories (*n* = 1) • Quebec (*n* = 1)	• Not available
Partnership Discussions	25	• CIHR Institutes (*n* = 12) • Provincial health authorities (*n* = 8) • Pan-Canadian organizations (*n* = 3) • International funding agencies (*n* = 2)	• Ontario (*n* = 6) • Quebec (*n* = 3) • British Columbia (*n* = 2) • Alberta (*n* = 2) • Saskatchewan (*n* = 1) • Manitoba (*n* = 1) • New Brunswick (*n* = 1) • Newfoundland (*n* = 1) • Yukon (*n* = 1) • US (*n* = 1) • Australia (*n* = 1)	• Not Available
IAB Discussions	14	• Researcher (*n* = 6) • Decision maker (*n* = 5) • Researcher/Healthcare provider (*n* = 1) • PWLLE (*n* = 2)	• Ontario (*n* = 5) • Quebec (*n* = 2) • British Columbia (*n* = 1) • Alberta (*n* = 1) • Saskatchewan (*n* = 1) • Manitoba (*n* = 2) • Nova Scotia (*n* = 1) • International (*n* = 1)	• Female (10) • Male (4)

^*^Interest-holder types can overlap, for example a key informant can give perspectives as a healthcare provider and researcher

^†^Geographical data for focus group participants is not reported because some participants shared perspectives representative of pan-Canadian challenges with integrated care whereas others did not report geographical location

### Retrospective analysis

The retrospective analysis of CIHR investments found that $75 M was invested over the period 2015–2020, including a total of 150 grants across four different funding programs (Health System Impact Fellowship, *n* = 21, Transitions in Care, *n* = 45, Community-based Primary Healthcare, *n* = 34, Strategies for Patient Oriented Research in Primary and Integrated Health Care Innovations Network, *n* = 50). The analysis revealed a gap in attention to the macro, policy, and system-level issues pertaining to the financing, funding, governance, organization, and delivery of integrated care. Past investments were often made in disease-specific domains for selective population sub-groups rather than for people with multiple chronic conditions or those at risk of poorer health outcomes due to structural and social determinants of health.

### Environmental scan

The environmental scan comprised 47 peer-review (*n* = 41) and grey literature (*n* = 6) articles published between 2011–2021. The websites of Canadian policy and partner organizations (*n* = 71) and funding agencies across Canada (*n* = 12) and internationally (*n* = 14) were also scanned to identify research priorities related to integrated care. The scan resulted in the identification of a core suite of elements essential to achieving transformative integrated care (described below).

Combined, this multi-method qualitative approach to designing a research funding initiative resulted in a shared view on integrated care transformation, priority areas and populations for research investment, initiative design elements to maximize research impact, and interest from multiple partners to support evidence-informed integrated care transformation.

### What we learned

#### A shared view on integrated care transformation: shifting towards evidence-informed solutions

There was a strong consensus in the community engagements to focus on the implementation, adaptation, and spread and scale of evidence-informed integrated care interventions and the need to shift away from the development and piloting of new integrated care innovations towards the implementation of promising interventions (i.e., those with an existing evidence base that supports intervention effectiveness) (Table 2). Both the environmental scan and community engagement reinforced the perceived need for transformative integrated care and highlighted a critical gap in understanding how to equitably implement and adapt evidence-based integrated care interventions/policies across different settings. This shift away from piloting new innovations was considered important in reaching new communities and populations in need and facilitating the growth and scalability of effective integrated care models for better overall performance of Canada's health care systems.

**Table 2:** Quotes illustrating key themes emerging from analysis

Key theme	Illustrative Quote(s)
Shift away from pilots towards developed, tested solutions (e.g., lines 300–304)	“(Integrated care needs) …. Working in partners in system who are invested in sustainable integrated care, not just a pilot…” – ***KII, Academic***
	“(Integrated care can add value by focusing on) …Successful models that have shown promise…” – ***Focus group, Healthcare provider***
	“How can we stop pilot paralysis? Funding implementation?…”—***Workshop. Academic***
Knowing what to do but not know how to do it (e.g., lines 310–311)	“…To provide effective support to leaders and managers to create sustainable integrated care programs, we need to understand how to effectively integrate programs.” ***– KII, Academic***
	“… if we don’t know how to implement them we don’t know how to show value…” ***– KII, Academic***
	“…Implementation science in integrated care is weak so implementation is a particular issue. Need to understand why certain programs have been successful (is it the culture? Formulation of team? How it was led?). There are many technical components, but a lot of success is because of relationships (mutual gain)…” ***– KII, Academic***
Community-based and led solutions (e.g., line 316–317)	“Community is the anchor of the iceberg …(we need) research to push through incorrect assumptions.. what actually works in community context and how we can support and nourish community as the home for integrated care. locally grounded equity lens…”*** – KII, Academic/Healthcare Provider***
	“Has to be community based, chronic disease is individual based but transformational effect is different …for example, rural areas, indigenous communities… they would gain the most from integrated care but fall through” ***– KII, Academic***
	“If the focus is on rapid translation of evidence, partners have to involve the communities you serve***” –KII, Academic***
	“Give reins to community to take some responsibility too – community can help orient the providers; invite them to community events; give understanding of social context and cultural context in which they’re practicing” ***– KII, Healthcare Provider***
	“Community engagement and community development. Must be done with Canadians, not for Canadians….” ***– Focus Group, Healthcare Provider***
The importance of patient-oriented research and co-design (e.g., line 316–317)	“…. missing links in Canadian health system is patient-led research. Pay attention to patient led research. Flip the classroom, for patients to lead research question.” ***– Focus group, Patient***
	“I also think patient-oriented research is critical for the area of integrated care. When we worry about integration at the level of professionals or systems, the benefits of those initiatives do not necessarily trickle down to result in better experiences or outcomes for patients.” ***– Workshop, Academic***
	“Co-design is very important in evaluation & impact, but must let them take the ownership of the implementation & evaluation.. they choose the measures that are important, rather than dictating what should be measured … helping them evaluate what they want evaluate … letting them fail… but there’s learning in failure too” ***– KII, Academic***
Shared leadership model (e.g., lines 360–363)	“The ground has shifted –…Within our network structure, everything we do has shared leadership e.g. medical lead, the shared leadership model” ***KII, Decision-maker***
	“Meaningful engagement can only happen with the right people at the table. Nice to have articulate people that can speak our language, but I believe that we will get richer information from the everyday people that struggle with healthcare, social care, isolation, homelessness etc.…’ ***Workshop, Decision-maker***
	“We need: shared purpose/vision, strong leadership, information sharing, fidelity, evaluation…To do this, we need regional supports and policy makers to make the space” ***– KII, Academic***
	“What can we learn from other jurisdictions about the funding models that would incent the ideal focus on integration to achieve outcomes over a longer time horizon?” ***– Workshop, Decision-maker***

The sentiment that 'we know what to do but not how to do it’ was frequently raised in key informant interviews and focus group discussions (Table 2). As a result, solution-oriented research emerged as a key lever for integrated care transformation. Research approaches such as implementation science, embedded research, integrated knowledge translation, policy research, and rapid response were identified as solution-oriented and essential in overcoming implementation and transformation challenges within complex health systems affected by political, social, economic and contextual factors.

The environmental scan, interviews and focus groups identified the importance of community-based and led solutions, underscoring the importance of patient-oriented research and co-design (Table 2). The use of learning health systems framework where data and evidence are routinely used to inform continuous improvements and where researchers work hand-in-hand with providers, decision-makers, and patients throughout the research and implementation process was also perceived as essential to advancing transformative integrated care.

#### Investing with a focus on the Quintuple Aim and priority populations

Community engagements highlighted the importance of aligning integrated care research and KM efforts towards a shared transformative purpose focused on the Quintuple Aim. Community engagements emphasized the importance of focusing investment on population groups most likely to benefit from integrated care, including:

- People living with complex health conditions (e.g., multiple chronic conditions, mental health, and substance use).
- Those affected by structural and social determinants of health, including Indigenous Peoples and marginalized communities (e.g., racialized communities, recent immigrants, individuals isolated geographically).

This is also illustrated by the following quote: *“…Look at the populations which are high cost, high user patients such as mental health, multiple chronic diseases…”(Academic researcher).*

#### Initiative design elements: co-leadership, intersectoral and cross-jurisdictional collaboration, collective impact

To drive meaningful impact, a critical design element for the initiative was investing in intersectoral and multi-jurisdictional teams for cross-jurisdictional learning, enhanced possibility of spread, greater potential for system level change, and to recognize the importance of integrated models of health and social care. KIIs highlighted the importance of multi-sectoral and cross-jurisdictional partnerships. A key informant stated *“…(For) Transformative impact – (we) need population-focused systems, not siloed disease focus programs. When integrated care moves beyond medical (to social and justice systems) it can become transformative…(With a) strong focus on inequities…” (Academic researcher).*

The importance of shared leadership was highlighted, with a strong belief expressed that transformation would not occur with teams comprised solely of researchers (Table 2). For example, the following quotes illustrate the need for active engagement by patient partners highlighted in the KIIs: *“Give reins to community to take some responsibility too – community can help orient the providers; invite them to community events; give understanding of social context and cultural context in which they’re practicing…” (Healthcare provider).* Similarly, another KII stated: *“Unless you have patient partners on the teams doing the research, I would make it a pre-req for researchers to have a patient on the team – having two patients involved is my goal and hope it is achievable…It is all about empowering the patient….” (Patient Partner).*

As a result, THINC required the leadership and composition of research teams to include providers, policy and decision-makers, people/communities with lived and living experience, and researchers. This quadripartite leadership model is designed to support active participation, meaningful involvement, and different perspectives throughout the research decision-making process. For example, providers can help inform the practical implementation of an evidence-informed intervention in a clinical setting, a policy or decision-maker can help align the research goals to the evidence needs of decision-makers and their policy levers and PWLLE can help ensure the research is patient-centred and reflective of the lived experience of those who will ultimately benefit from the intervention/policy.

A shared leadership model was perceived as key to understanding and incorporating the real-world priorities and contexts of those shaping, delivering, and receiving integrated care, as well as advancing the successful, adapted implementation, scale and spread of evidence-informed solutions within new populations, settings, and contexts.

Several additional important considerations emerged including how to effectively align a variety of distinct funding opportunities and research projects towards a shared goal of advancing transformative integrated care, how to meaningfully support and enhance collective efforts of KM and impact, and how to ensure that peer review criteria appropriately recognizes and rewards collaborative, community-engaged, solution-oriented research.

### What we built: the THINC initiative

The CIHR-IHSPR team commenced the initiative design process with a commitment to focus on integrated care, informed by its prior strategic planning engagements. However, where to focus within the broad scope of integrated care, what types of research were most needed, and how to design an impactful and transformative initiative were unknown. The evidence-informed, community-engaged initiative development process described above was designed to elicit evidence and insight to directly inform the objectives, scope, and key design elements of the initiative. The resulting initiative, *Transforming Health with Integrated Care (THINC*), aimed to improve the understanding of how to implement, evaluate, adapt and/or spread and scale evidence-informed integrated care policies and interventions within and/or beyond the formal health care delivery system towards advancing the Quintuple Aim.

THINC focuses on the priority populations that were identified through the community engagements and environmental scan: people living with complex health needs (e.g., multimorbidity, mental health needs and/or substance use) and those currently living with or at risk of experiencing poor health outcomes based on social economic factors, and/or those identifying with historically underrepresented populations (e.g., racialized communities, people living with disabilities, LGBTQIA/2S, Indigenous Peoples) (Imagine Canada, 2022). Funding pools specific to rural, remote, northern communities, Indigenous Peoples, equitable, diverse and inclusive integrated care models were included as design elements.

THINC is comprised of a suite of strategic funding opportunities designed to advance evidence-informed, integrated care across Canada. This suite encompasses research approaches that were identified as enablers of solution-oriented research impact in the community engagements and environmental scan, including implementation science, embedded research, learning health systems, policy research, patient-oriented research, KM, and rapid response research. Each funding opportunity aligns the research to the priorities of importance to the health system (i.e., to the knowledge user and health system organization partners), and to the community by requiring that patient/community needs and experiences are central to the research and implementation approach. Each funding opportunity incorporates a co-design approach where researchers, decision-makers, healthcare providers, and PWLLE (or some combination of) are required to work together throughout the research process. This shared leadership model aims to ensure meaningful involvement of all types of interest-holders in shaping the research from the outset, i.e. defining the research question, methodologies, strategies. Equity is centred as a key outcome of importance in all the funding opportunities via a focus on advancing the Quintuple Aim. The peer review criteria reflect a concept of research excellence that values scientific merit, shared leadership and collaboration, and the impact potential of the research. Additionally, through partnership with SPOR, the THINC initiative incorporates patient-oriented research and integrated knowledge translation (iKT) as fundamental design elements requiring co-design and co-production throughout the research and KM process.

THINC’s suite of funding opportunities includes: 5-year implementation science team (IST) grants; 1-year policy research and Quadruple Aim catalyst grants; embedded research awards; and a KM and Impact Hub that is designed to support and connect all teams and awardees funded by THINC, build relationships with system leaders grappling with challenges related to integrated care and support the mobilization of the resulting research for real-world impact (see Table 3 for details).

**Table 3:** THINC funding initiative eligibility and application review criteria: an overview

Program Component	Program Information	Program Metrics
Hub – Knowledge Mobilization and Impact Hub (learn more)	**Objectives:** Foster initiative wide coordination, learning and collaboration, capacity development, KM and collective impact to advance integrated care transformation	**Grant Value**: $2.2 M **Duration**: 6 years **Partners**: 2 **Total Investment**: $2.2 M **Projects Funded**: 1
	**Eligibility Criteria:** Hub team must include: • Scientific Lead • Knowledge Mobilization and Brokering and Impact Lead • Community Engagement Champions • Individuals with lived/living experiences related to integrated care • Sex and Gender • Indigenous Health Research and Equity, Diversity and Inclusion champions (learn more)	
	**Review Criteria:** Research approach must include: • Originality and value-add of the proposal • Applicant team, including diversity, qualifications and expertise of team members, • Environment for research, including suitability for hub’s activities and training and mentoring • Impact of the research, geography, training, expertise and a meaningful engagement plan for overall Hub activities (learn more)	
Teams – Implementation Science Teams (learn more)	**Objectives:** Advance the implementation, evaluation, and spread/scale of transformative evidence-informed integrated care policies and interventions towards advancing the Quintuple Aim	**Grant Value**: $2 M **Duration**: 5 years **Partners**: 11 **Total Investment**: $24.4 M **Projects Funded**: 13
	**Eligibility**: Core leadership team must include: • Quadripartite leadership model (researcher, decisionmaker, healthcare provider, person with lived/living experience) Sex and gender champion • Equity, diversity and inclusion champion (learn more)	
	**Review Criteria:** • Equity, diversity and inclusion in all research stages • Experience in patient-oriented care, meaningful engagement with knowledge users of the research • Interdisciplinary, multisectoral collaborations (learn more)	
Catalyst Grants 1—Accelerate Evidence on Healthcare Delivery System Innovations that Achieve the Quadruple Aim and Improve Health Equity (learn more)	**Objectives**: Support Knowledge Creation and Knowledge Implementation projects to generate relevant and timely evidence about the impacts of innovations in how healthcare systems and services are organized, delivered, governed, held accountable, financed, and/or funded on the Quintuple Aim, and which innovations have the most potential for transformative impact in the Canadian context	**Grant Value**: $100,000 **Duration**: 1 year **Partners**: 6 **Total Investment**: $4.9 M ($1.8 M focused on integrated care) **Projects Funded:** 50 (18 focused on integrated care)
	**Eligibility Criteria**: Research must be co-led by: • Researcher and a knowledge user with direct experience in the research area (learn more)	
	**Review Criteria:** • Meaningful collaboration of knowledge users as part of the research team • Alignment with priority evidence-needs • Plans for integrated knowledge translation plans • Future collaborative partnership opportunities (learn more)	
Catalyst Grants 2 – Policy Research for Health System Transformation (learn more)	**Objectives**: Support retrospective policy evaluation and/or prospective policy development and implementation that generates evidence to inform macro-level policies to support high-performing health care and public health systems that advance the Quintuple Aim	**Grant Value**: $150,000 **Duration**: 1 year **Partners**: 13 **Total Investment**: $9 M ($2.25 M focused on integrated care) **Projects Funded:** 60 (15 focused on integrated care)
	**Eligibility Criteria:** Research team must include: • tripartite leadership researcher, policymaker, and person with lived experience) (learn more)	
	• **Review Criteria**: Integrated knowledge translation approach • Diverse disciplinary expertise • Meaningful engagement and commitment from the research team • Contributions of all applicant partners in advancing the research objectives (learn more)	
Embedded Research – Health System Impact Fellowships and Embedded Early Career Researcher Awards (learn more)	**Objectives**: Grow a strong cadre of embedded researchers positioned to play a key role in evidence-informed health system improvement that advances the Quintuple Aim	**Grant Value**: Varies by career stage **Duration**: Varies by career stage **Partners**: 13 + **Total Investment**: $45 M over 8 years **Projects Funded:** Varies by career stage
	**Eligibility Criteria**: Applicant must • Be a doctoral trainee, postdoctoral researcher, or early career researcher • Have a dyad supervisor/mentor model including a health system supervisor/mentor as an executive level decision-maker within a health system organization and an academic at a Canadian university (learn more)	
	**Review Criteria**: • Applicant – Experience, Contributions and Career Development • Research Program and Knowledge Mobilization Plan • Environment, Support and Mentorship • Potential Impact (including value-add of the award to the individual and organization) (learn more)	

Though the funding opportunities share the core design elements outlined above, they each have distinct strategic objectives informed by the community engagements. For example, a historical focus of CIHR investments in disease-specific domains illuminated a gap in research focused on the macro, policy, and system-level issues related to the financing, funding, governance, organization, and delivery of integrated care. This helped inform the focus of the Policy Research for Health System Transformation catalyst grant, which aims to support research to inform the development and implementation of macro-level health system policies [57]. Similarly, to move beyond small-scale, short duration pilot projects towards the implementation and spread of proven/promising solutions, the THINC ISTs are grounded in implementation science, require a focus on *‘transformative evidence-informed integrated care policies and intervention(s) that have been developed, piloted, tested and/or evaluated elsewhere with published evidence of effectiveness (and/or promising results on improved integration of services and improved outcomes,*’ and have clear impact goal(s) grounded in the Quintuple Aim with measurement and outcomes for evaluation [58]. To recognize the importance of providing dedicated support for KM and impact, aligning funding opportunities towards a shared goal, and building community, capability, and collaboration across programs, a KM and Impact Hub was incorporated as a foundational initiative component. The THINC Hub was designed to “build and support a vibrant pan-Canadian learning community involving all THINC grantees and knowledge user communities towards advancing evidence-informed integrated care transformation and catalyze progress towards achieving the Quintuple Aim [59]”.

Partner engagements throughout the initiative design process revealed a shared commitment to co-investing to support evidence-informed integrated care transformation. This resulted in funding partnerships with multiple provincial and federal organizations that increased the scale and scope of initiative and the number of grants and awards that could be supported. Overall, CIHR-IHSPR and partners mobilized more than $30 million for the THINC initiative.

## Discussion

This paper presents a health research funding organization’s perspective on designing and developing a large-scale, solution-oriented funding initiative—*Transforming Health with Integrated Care (THINC)—*using a comprehensive, evidence-informed, community-engaged, approach. This approach informed the initiative’s aims, research funding areas and strategies, contributing to the science of funding research impact.

### Funding science for real-world impact – the role of research funders

Health research funders’ roles extend beyond funding to include monitoring outcomes, championing evidence-use, and mobilizing research evidence [60]. McLean et al. found that funders increasingly prioritize knowledge translation (KT), e.g., within their mandate or funding calls [61]. In the US, the Veterans Affairs’ *Office of Research and Development* (ORD) Research Lifecycle emphasizes promising innovations, rapid response, implementation science, capacity development and impact and evaluation, similar to THINC [62]. While the understanding of funding strategies optimizing real-world impact remains limited, this study provides evidence on effective approaches.

### Prioritizing populations who can benefit the most from transformative integrated care

Integrated care can address unmet health-related social needs of people with complex care needs [63]. A key priority in social determinants of health (SDOH) research is broadening funding beyond single-disease approaches [64, 65]. Beidas et al. [65] emphasized that enhancing real-world impact requires including upstream social and structural determinants of health [65]. THINC prioritizes individuals with complex care needs and those experiencing or at risk of poor outcomes due to social determinants of health, tackling root causes of health disparities for equity-centered impact.

### Implementation science and embedded research to advance real-word impact

Implementation science and embedded research are recognized strategies for advancing real-world impact [66, 67] by integrating evidence-based interventions into real-world settings with interdisciplinary collaboration. These methodologies identify implementation barriers and enablers in dynamic, real-world settings [68]. THINC ISTs inform the implementation, evaluation, adaptation and/or spread/scale of integrated care policies and interventions using learning health system, patient-oriented approaches with rapid learning and KM strategies incorporated throughout the research process. The teams are led by a quadripartite leadership model (researcher, decision-maker, health provider, PWLLE), collectively accountable for planning and developing shared pathways towards impact.

Embedded research aligns research with health system needs, enhancing research relevance, improving rapid learning, and fostering trusted relationships [42, 69]. Kitzman et al. [42] found embedded research drives value-based care [42]. THINC’s embedded research fellowships provide PhD trainees, postdoctoral researchers, and early career researchers with the embedded research opportunities to address pressing integrated care challenges in health system organizations [70].

### Collaboration, co-design, and co-leadership

Collaboration and co-design are key to advancing real-world impact [71]. An international study of 13 funding agencies’ report that despite most funders (~ 65%) emphasizing science integration into policy and practice, only 23% used needs-based funding mechanisms including co-creation of evidence with knowledge users [40]. The community-engaged approach to designing the THINC initiative demonstrates the feasibility and value of engaging with multiple perspectives to inform the scope (e.g., promising integrated care solutions, priority populations) and funding design elements (e.g., need for community-based and community-led solutions, rapid response approaches and on-going engagement activities).

Collaboration and co-design were determined essential design elements for the research to achieve transformative integrated care. A recent review identified meaningful community engagement and co-design approaches throughout the research process as critical for effective action and desired outcomes [41]. Similarly, co-leadership in integrated health and social care research has shown to support well-informed decision-making, continued learning and a sense of shared responsibility [72]. THINC requires grantees to co-lead research through a co-leadership model involving research, policy/practice, and lived experience.

### Strengths and limitations

This study uses an evidence-informed, community-engaged approach with triangulation of methods and data sources for validity; and mobilization of partnerships to increase the resources for and scope of the initiative. Limitations include lack of detailed participant demographic data of participants and English-only review and engagement, though efforts were made to ensure diverse perspective. Recruitment was conducted through purposive and snowballing sampling, which may have introduced biases in participant selection, potentially limiting diversity in perspectives. Furthermore, biases in the analysis could have arisen from the subjective nature of qualitative data interpretation, although efforts were made to mitigate this risk through team discussions.

Comprehensive community engagement and environmental scans require substantial resources, posing challenges for some funders. Future evaluation of longitudinal outcomes and impacts of initiatives like THINC can improve our understanding of the effectiveness and impacts of certain funding design elements and strategies.

## Conclusion

Research funders can enhance real-world impact by prioritizing applied research, using solution-oriented research strategies (e.g. implementation science, embedded research), rewarding partnerships, monitoring research impacts on practice, health, and policy outcomes, and contributing to the science of science literature [10]. Evidence-informed and community-engaged approaches in health research improve quality, relevance and uptake of research, however there is a lack of understanding on how these strategies can be applied in the design of research funding initiatives [73].

This paper addresses this gap by presenting an evidence-informed, community-engaged approach to the design of the THINC research initiative, a large-scale strategic research initiative focused on transformative integrated care. THINC aims to advance Canada’s achievement of the Quintuple Aim and embodies the principles of implementation science by fostering interest-holder buy-in, co-creating shared objectives, generating shared interests, and infusing implementation science methodologies into its flagship program, THINC ISTs. By shifting the research focus towards already tested and promising interventions, THINC contributes to the acceleration of adoption of proven integrated care models across Canada.

Furthermore, this study underscores the significance of integrating community perspectives through multiple methods to design research funding opportunities aligning with real-world needs and challenges. Research strategies such as implementation science, embedded research, and KM including the THINC KM & Impact Hub as an initiative component, and design elements such as co-leadership, cross-jurisdictional, interdisciplinary teams are highlighted as critical components intended to enhance the relevance and impact of the research funded. This paper illustrates how a comprehensive, evidence-informed, community-engaged approach can inform the design of a large-scale, solution-oriented, transformational research funding initiative and, through the process, stimulate collaborative partnerships with a shared purpose and commitment to work together to fund for impact.

## Supplementary Information

Supplementary Material 1.

Supplementary Material 2.

Supplementary Material 3.

## Data Availability

The datasets used and/or analyzed during the current study are available from the corresponding author on reasonable request.

## Abbreviations

CBPHC: Community-based Primary Health Care
CHSPRA: Canadian Health Services and Policy Research Alliance
CIHR: Canadian Institutes of Health Research
CIHR IHSPR: Canadian Institutes of Health Research Institute of Health Services and Policy Research
COVID-19: Coronavirus Disease of 2019
DORA: Declaration of Research Assessment
HSPR: Health Services and Policy Research
KM: Knowledge Mobilization
KT: Knowledge Translation
LGBTQIA/2S: Lesbian, Gay, Bisexual, Transgender, Questioning, Intersex, Asexual and Two-Spirit
NHS: National Health Service
NIHR: National Institute for Health and Care Research
PWLLE: People with lived and living experience
RIA: Research Impact Analysis
SPOR: Strategies for Patient-Oriented Research Network
THINC: Transforming Health with Integrated Care
TiC: Transitions in Care (TiC) initiative
UK: United Kingdom
US: United States

## Footnotes

1 Interest-holders engaged: policy and decision-makers, healthcare providers, patients and community members, and researchers.

2 In the context of this paper, person with lived and/or living experience (PWLLE) refers to patients, family members, caregivers, community members with experience with integrated or fragmented, uncoordinated care.
