# Supplementary material for: An evidence-informed, community-engaged approach to designing a large-scale, impact-oriented research funding initiative to foster the implementation of transformative integrated care: a multi-methods qualitative study
Source: Implement Sci Commun. 2025 Aug 15;6:86. doi: 10.1186/s43058-025-00760-7 (PMC12355821; doi:10.1186/s43058-025-00760-7)
Supplement: Supplementary file 1 — Supplementary Material 1. [file 43058_2025_760_MOESM1_ESM.docx]

## Appendix 1. THINC Initiative: Key-Informant Interview Guide

### Stream 1: Understanding transformative integrated care and the building blocks essential to achieving it

1. We are working towards developing a common definition of transformative integrated care. Integrated care is, oftentimes, synonymously used with terms such as ‘*continuity of care’* or ‘*transitions of care’*. What does ‘transformative integrated care’ mean to you?
   - ***Prompt***: For example, with transformative integrated care being integrated care that is transformative from both the patient and system perspectives (e.g. improved efficiencies).
2. Literature cites essential elements or ‘building blocks’ of high-performing practices to integrating care. In your experience, what are the critical elements or building blocks to achieving successful integrated care? These can be at the system/policy, community/organization, and/or provider/patient –levels, e.g. data platforms, digital health, Health Human Resources (HHR), aligned funding models.
   - ***Prompt***: What are some examples of levers and enablers *vs* risks and barriers to transformative integrated care using such building blocks? (e.g. virtual care, digital health, and types of technology used). Which is the most important?
   - ***Prompt*:** What are some examples of building blocks of integration successful within a certain group or context that can be transferrable to a wider context?

### Stream 2: Understanding the value-add of evidence and knowledge mobilization in transformative integrated care

1. Where and how can evidence and knowledge mobilization add the most value in transforming healthcare systems towards integrated care? What type of research is required to improve integrated care (e.g. development evaluation, rapid learning, implementation science, methods development, evaluation analysis, impact assessment)?
   - ***Prompt***: What are the key evidence needs to integrating care?
2. What should a research investment in transformative integrated care aim to achieve? What do you see as the overarching goals?

### Stream 3: Understanding the impacts of integration

1. What are some key examples of successful and transformative integration of care (e.g. care processes, digital health technologies), and what were some of the key impacts and outcomes (e.g. patient care, experience, cost)?
   - ***Prompt***: What are the key features (of the models of integration)? What did, or will it achieve, and from whose perspective?
   - ***Prompt***: How can health equity be achieved by integrating care? Can you share some examples?
2. Various stakeholders (providers, policymakers, communities) play a role in shaping the integration of care. When you think about the examples of successful integration that you shared, who are the key partners we (CIHR) should be engaging with to advance and sustain transformative integrated care, and why?
   - ***Prompt***: How can we (CIHR) ensure strong and continuing engagement between research teams and the partners you identified?

### Anticipated results

Interviews conducted will inform ongoing and future development of funding opportunities and initiatives. More specifically, we will gain an improved understanding of elements necessary for the integration of care, the role of evidence, the impacts of integrated care, how and why they arise, and how best to showcase them.
