# Supplementary material for: An evidence-informed, community-engaged approach to designing a large-scale, impact-oriented research funding initiative to foster the implementation of transformative integrated care: a multi-methods qualitative study
Source: Implement Sci Commun. 2025 Aug 15;6:86. doi: 10.1186/s43058-025-00760-7 (PMC12355821; doi:10.1186/s43058-025-00760-7)
Supplement: Supplementary file 2 — Supplementary Material 2. [file 43058_2025_760_MOESM2_ESM.docx]

## Appendix 2. THINC Initiative: Focus Group Discussion Guide

### Patient and Public Community members

#### Successful Integration

1. **What does successfully integrated care mean to you? Are there examples you can share by experience, or how you thought you would have liked to receive integrated care?**
   1. **From your perspective, what is the role of evidence in achieving transformative integrated care? What should we (CIHR) prioritize when funding research in integrated care?**

#### Role of Research

1. **How can we (CIHR) support meaningful, and inclusive engagement between researchers, the public, and patient communities?**
   1. **What are some effective ways to engage with a group like this?**

### Health and Social care Providers

#### Successful Integration

1. What does successfully integrated care **mean to you? Integrated care** is oftentimes referred to as ‘continuity of care’ or ‘transitions of care’.
   1. How does integration **add value** to the care you provide (relative to non-integrated care)?
   2. Can you point to any **examples** of successful integrated care? Or examples of failed integration of care and ideas for how the situation can be improved?
2. Literature cites essential elements or ‘**building blocks’** to successfully integrating care. In your experience, what are the critical elements or building blocks that support you in providing integrated care? *For example, organizational policies that influence how you provide care, the type of digital infrastructure available to you to access and share a patient’s health records, etc.*

#### Role of Research

1. In which areas and how can research bring the most value in supporting and advancing the coordination and delivery of integrated care?
   1. In your view, are there specific populations that should be targeted for priority investment in the area of integrated care research?
   2. How can we (CIHR) as a research funding agency support the design, implementation, and spread of integrated care solutions that will **ensure inclusivity and equity** of those receiving and/or providing care?
2. How can we (CIHR) **support meaningful engagement** between researchers and the health and social care provider community to create and mobilize relevant, useful, and timely evidence?

### Rural Health Perspectives

#### Successful Integration

1. **What does successfully integrated care mean to you? Integrated care is oftentimes referred to as ‘continuity of care’ or ‘transitions of care’.**
   1. Can you share examples of existing innovative models of integrating care in the rural context?
   2. Are there examples of (potential or made) impacts of integrating care within the rural context?
2. Literature cites essential elements or ‘**building blocks’** to successfully integrating care. In your experience, **what are the critical elements or building blocks to integrating care for rural populations? *For example, meaningful engagement of rural communities, coordination of services within and across sectors, or strengthening governance and accountability of rural health organizations.***
   1. **Are there examples of building blocks critical in the rural context that are also transferrable to other health system settings?**

#### Role of Research

1. **How do you think we (CIHR) can work to move the needle in the right direction (towards successful integrated care)? In other words, what should we prioritize when funding research in integrated care in the rural context?**
   1. **How can we (CIHR) as a research funding agency support the design, implementation, and spread of integrated care solutions that will ensure inclusivity and equity of those receiving and/or providing care in the rural context?**
2. **How can we (CIHR) support meaningful and inclusive engagement between researchers and patient and provider communities to create and mobilize relevant, useful, and timely evidence needed to advance integrated care in the rural context?**
   1. **Are there mechanisms to ensure accountability towards successfully integrating care in the rural context?**

### Exit Question (for all focus group discussions)

1. **Is there anything else you would like to share on how integrated care models can be improved in rural Canada?**
