# Supplementary material for: An evidence-informed, community-engaged approach to designing a large-scale, impact-oriented research funding initiative to foster the implementation of transformative integrated care: a multi-methods qualitative study
Source: Implement Sci Commun. 2025 Aug 15;6:86. doi: 10.1186/s43058-025-00760-7 (PMC12355821; doi:10.1186/s43058-025-00760-7)
Supplement: Supplementary file 3 — Supplementary Material 3. [file 43058_2025_760_MOESM3_ESM.docx]

## Appendix 3. THINC Initiative: Virtual Workshop Discussion Guide

### Successful Integration

Panel question:

- What does **successfully integrated care** mean to you? What are some **examples** of successful integrated care models?

Audience questions:

- - What does **successfully integrated care** mean to you?
  - What is an ‘ideal’ experience interacting with multiple healthcare providers and/or across different sectors?

### Role of Research

Panel question:

How can **research best support** the coordination and delivery of **integrated care** that is inclusive of all Canadians while improving care and experiences, cost, efficiency, and equity?

Audience questions**:**

- What are some promising approaches to foster meaningful engagement of patients, providers, and decision makers in integrated care research (note: please answer from your perspective as a patient, caregiver, provider, decision maker, researcher or other)?
- Are there certain issues within integrated care that should be prioritized for research investment? What are they and why?
